# The first progress of plasma-based transition metal dichalcogenide synthesis: a stable 1T phase and promising applications

**DOI:** 10.1039/d1na00882j

**Published:** 2022-04-25

**Authors:** Hyeong-U. Kim, Hyunho Seok, Woo Seok Kang, Taesung Kim

**Affiliations:** Department of Plasma Engineering, Korea Institute of Machinery & Materials (KIMM) Daejeon 34103 Korea; SKKU Advanced Institute of Nanotechnology (SAINT), Sungkyunkwan University Suwon 16419 Korea tkim@skku.edu; School of Mechanical Engineering, Sungkyunkwan University Suwon 16419 Korea

## Abstract

Two-dimensional (2D) transition metal dichalcogenides (TMDs) have attracted attention as polymorphs depending on their phases (1T and 2H) when applying typical synthesis methods. The 2H phase is generally synthesised through chemical vapour deposition (CVD) on a wafer-scale at high temperatures, and many synthesis methods have been reported owing to their thermodynamic stability and semiconductor properties. By contrast, although the 1T phase is meta-stable with an octahedral coordination, thereby limiting the use of synthesis methods, the recent structural advantage in terms of the hydrogen evolution reaction (HER) has been emphasised. Despite this demand, no large-area thin-film synthesis method for 1T-TMDs has been developed. Among several strategies of synthesizing metallic-phase (1T) TMDs, chemical exfoliation (alkali metal intercalation) is a major strategy and others have been used for electron-beam irradiation, laser irradiation, defects, plasma hot electron transfer, and mechanical strain. Therefore, we suggest an innovative synthesis method using plasma-enhanced CVD (PECVD) for both the 1T and 2H phases of TMDs (MoS_2_ and WS_2_). Because ions and radicals are accelerated to the substrate within the sheath region, a high-temperature source is not needed for vapour ionisation, and thus the process temperature can be significantly lowered (150 °C). Moreover, a 4-inch wafer-scale of a thin film is an advantage and can be synthesised on arbitrary substrates (SiO_2_/Si wafer, glassy carbon electrode, Teflon, and polyimide). Furthermore, the PECVD method was applied to TMD-graphene heterostructure films with a graphene-transferred substrate, and for the first time, sequential metal seed layer depositions of W (1 nm) and Mo (1 nm) were sulfurized to MoS_2_-WS_2_ vertical heterostructures with Ar + H_2_S plasma. We considered the prospects and challenges of the new PECVD method in the development of practical applications in next-generation integrated electronics, HER catalysts, and flexible biosensors.

## Abstract 2

An insight into the synthesis of 2-dimensional transition metal dichalcogenides (TMDs) and classification of the phases with various synthesis methods. Herein, the 1T phase of TMDs is reviewed with a unique synthetic method with cold plasma (plasma enhanced CVD).

## Graphical abstract

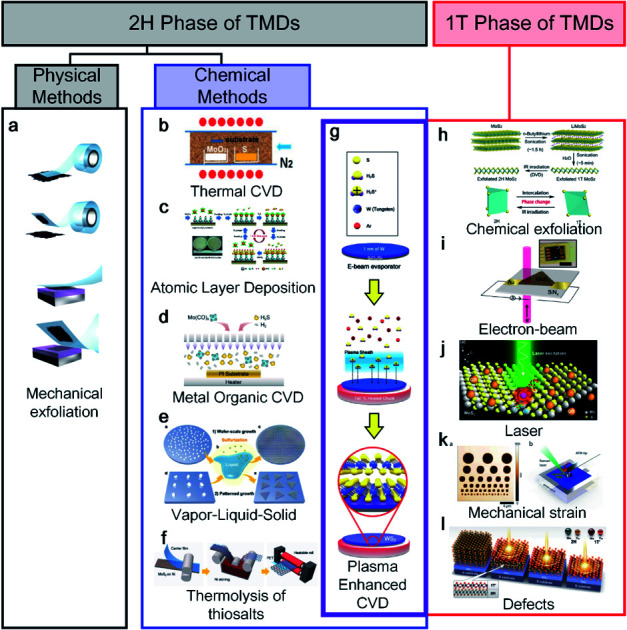

## 1. Introduction

The use of graphene exfoliation has rapidly increased in two-dimensional (2D) materials.^1^ Owing to a compounding effect, the 2D library has grown every year with more than 150 exotic 2D materials, including transition metal dichalcogenides (TMDs) with a chemical formula of MX_2_ (M: transition metal and X: chalcogen).^2^ Unlike graphene, TMDs with a bandgap are actively used in the semiconductor field. Molybdenum disulfide (MoS_2_) has a tunable bandgap depending on the number of layers, similar to that of conventional silicon-based semiconductors.^3^ Including MoS_2_, TMDs have unique properties as polymorphs depending on the phases, such as 1T (metal), 1T′ (semimetal-like), and 2H (semiconductor) phases. The 2H phase, for which many synthesis methods have been reported, is thermodynamically stable with the trigonal-prismatic coordination of the transition metal atoms, allowing researchers to extend it to various applications. However, the 1T phase is meta-stable with an octahedral coordination, and thus synthesis methods are limited, and are even naturally converted into a 2H phase.^4^

The phase transition from 2H to 1T by applied strain, defect engineering, and chemical intercalation can force the material into an intermediate state (1T′ phase) which is fully converted to the 1T phase.^5^ Liu *et al.* succeeded in phase-selective synthesis of 1T′-MoS_2_ with theoretical investigation and experimental confirmation by improved electrochemical performance compared to 2H-MoS_2_.^6^ Peng *et al.* demonstrated the direct synthesis of 1T′-MoS_2_ by chemical exfoliation comprising over 97% purity and millimeter-sized superconducting nanosheets.^7^ Also, a one-pot colloidal chemical process for stable 1T′-WS_2_ nanoparticles was established exhibiting superior HER performance to 2H-WS_2_.^8^

Herein, we succeeded in developing for the first time a plasma synthesis method using a synthesis mechanism. In 2015, 2H-MoS_2_ was first synthesised by plasma-enhanced chemical vapor deposition (PECVD)^9^ and then 1T-WS_2_ was also successfully synthesized in 2020^10^ with cold plasma. Finally, 1T-MoS_2_ was also synthesized in 2021.^11^ Unlike the existing synthesis methods, cold plasma was used as an energy source for TMD synthesis, and it can thus achieve an extremely low process temperature of 150 °C to 300 °C. Because we used a modified inductively coupled plasma (ICP, 13.56 MHz) type reactive ion etcher (RIE) machine, it has sufficient energy to penetrate the transition metal layer (*e.g.* molybdenum or tungsten) for sulfurization without the need for a high process temperature.

We then reviewed the classification of existing synthesis methods for the 2H and 1T phases of TMDs. The 2H phase has many synthesis methods that can be classified into mechanical and chemical methods. Furthermore, only the 2H phase can be synthesised at the wafer-scale, and thus it was reviewed separately. Finally, we introduced a new synthesis method using cold plasma, which is a unique method for both 1T and 2H phases with a 4-inch wafer-scale. The plasma method has a new mechanism with a nano-grain size of both phases, and it is therefore possible to stabilise the 1T phase which is metastable.

## 2. Classification of existing synthesis methods for 2H and 1T phases

**Fig. 1:**
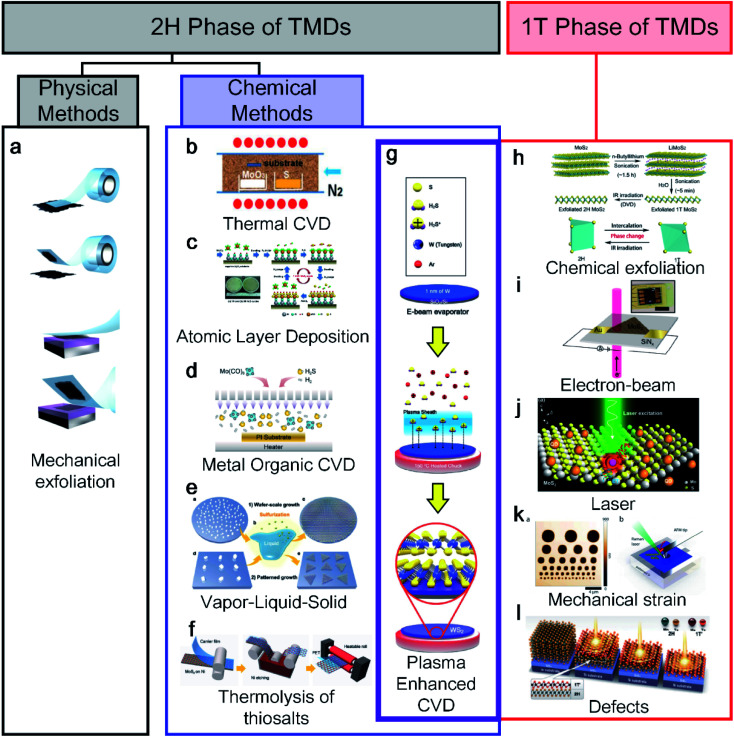
An overview diagram of a classification synthesis method for 2H and 1T phases of transition metal dichalcogenides (TMDs): (a) mechanical exfoliation (adapted with permission from ref. 12, copyright 2012 IOP Publishing), (b) CVD (adapted with permission from ref. 17, copyright 2012 Wiley-VCH Verlag GmbH & Co), (c) ALD (adapted with permission from ref. 18, copyright 2014 Royal Society of Chemistry), (d) MOCVD (adapted with permission from ref. 19, copyright 2019 American Chemical Society), (e) VLS (adapted with permission from ref. 21, copyright 2019 Royal Society of Chemistry), (f) thermolysis of thiosalts (adapted with permission from ref. 22, copyright 2017 Wiley-VCH Verlag GmbH & Co), and (g) plasma synthesis method for both 1T and 2H phases of TMDs (adapted with permission from ref. 10, copyright 2020 Wiley-VCH Verlag GmbH & Co). 1T phase of TMD synthesis methods: (h) chemical exfoliation (adapted with permission from ref. 24, copyright 2015 American Chemical Society), (i) electron beam (adapted with permission from ref. 28, copyright 2016 American Chemical Society), (j) laser (adapted with permission from ref. 29, copyright 2016 Springer), (k) mechanical strain (adapted with permission from ref. 30, copyright 2016 American Chemical Society), and (l) defects (adapted with permission from ref. 31, copyright 2015 American Association for the Advancement of Science).

TMDs have various structural polymorphs; therefore, they can be classified depending on the phases. First, a thermodynamically stable polymorph of the semiconducting 2H phase is common and easy to discover using physical methods. Novoselov and Geim *et al.* discovered the exfoliation of graphene from graphite using scotch tape in 2004,^1^ the method of which is described in Fig. 1a.^12^ Moreover, chemical vapor deposition (CVD) has received increased interest for use with graphene^13,14^ and other 2D materials.^15^ Bae *et al.* made CVD-graphene attractive for applications in flexible electronics through the roll-to-roll production of a 30-inch graphene film in 2010.^16^ After the successful CVD growth of graphene, Lee *et al.* also succeeded in synthesising a monolayer of MoS_2_ with MoO_3_ and S powder using CVD in 2012.^17^ However, the CVD method requires a high process temperature (650 °C to 1000 °C), making it difficult to use when a low-temperature or transfer process is required when applying an arbitrary substrate, as shown in Fig. 1b. As an alternative, atomic layer deposition (ALD)^18^ and metal–organic CVD (MOCVD),^19,20^ which are other chemical methods, have been used at relatively low temperatures (Fig. 1c and d). Another method is vapor–liquid–solid (VLS) growth involving molten precursors (non-volatile Na_2_MoO_4_) with temperatures higher than the melting point (687 °C), as shown in Fig. 1e.^21^ The thermolysis of thiosalt-based synthesis was reported along with a roll-to-roll transfer, as indicated in Fig. 1f.^22^ The thermal decomposition of (NH_4_)_2_MoS_4_ was converted into MoS_2_ with N_2_ at 600 °C. As shown in the crossed purple area in Fig. 1g, plasma-enhanced CVD (PECVD) has been to date the only way to synthesise both 1T and 2H phases.^10^

Unlike the 2H synthesis method, the 1T synthesis method is limited and relatively difficult to apply because it is metastable under ambient conditions. Therefore, the importance of the 1T phase has received attention relatively later than the 2H phase in various applications. In particular, the 1T phase has many S vacancies as active catalytic sites because its structure can be advantageous for the hydrogen evolution reaction as a catalyst.^23^ The most representative 1T synthesis method is chemical exfoliation using electrochemical Li^+^ intercalation (Fig. 1h^24^, including *n*-BuLi,^23,25^*t*-BuLi,^26^ and LiBH_4_ (ref. 27)). In general, the chemical exfoliation method is attained as small crystals ranging from 1 to 10 nm. Parkin *et al.* reported the use of phase engineering under electron irradiation with an S vacancy density, as shown in Fig. 1i.^28^ The electron beam is irradiated to a diameter of 2–12 μm, allowing only the 1T phase area to be localised. Similarly, a laser is irradiated to a localised area of the monolayer of MoS_2_ for transforming the 1T phase.^29^ Song *et al.* reported a phase transition from 2H to a 1T-MoTe_2_ film at room temperature by applying a tensile strain with an AFM tip, as shown in Fig. 1k.^30^ In addition, Cho *et al.* reported laser-induced phase patterning to fabricate an ohmic heterophase homojunction. Multilayer 2H-MoTe_2_ was used for phase patterning in the desired area, as shown in Fig. 1l.^31^

**Fig. 2:**
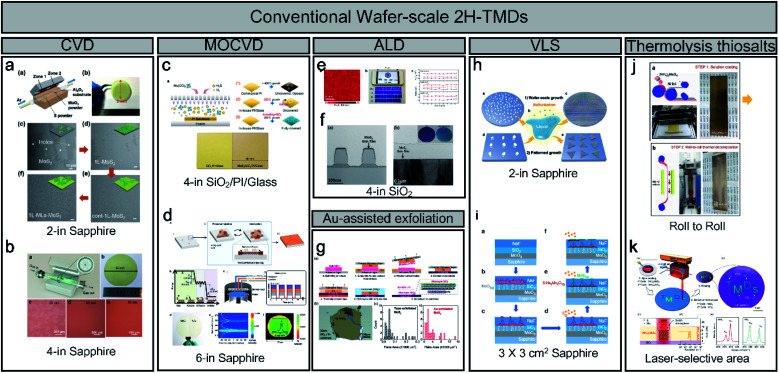
An overview of conventional wafer-scale 2H-TMDs: (a and b) chemical vapor deposition (CVD) (adapted with permission from ref. 38, copyright 2018 Wiley-VCH Verlag GmbH & Co, and from ref. 39, copyright 2020 American Chemical Society), (c and d) MOCVD (adapted with permission from ref. 19, copyright 2019 American Chemical Society, and from ref. 32, copyright 2020 Wiley-VCH Verlag GmbH & Co), (e and f) atomic layer deposition (ALD) (adapted with permission from ref. 33, copyright 2016 Nature Publishing Group, and from ref. 34, copyright 2016 Elsevier), (g) Au-assisted exfoliation (adapted with permission from ref. 35, copyright 2016 Wiley-VCH Verlag GmbH & Co), (h and i) vapour–liquid–solid (VLS) (adapted with permission from ref. 21, copyright 2019 Nature Publishing Group, and from ref. 36, copyright 2020 Royal Society of Chemistry), and (j and k) thermolysis of thiosalts (adapted with permission from ref. 22, copyright 2017 American Chemical Society, and from ref. 37, copyright 2020 American Chemical Society).

Unlike the method-limited 1T phase, 2H synthesis methods have been well developed and have realised wafer-scale monolayer MoS_2_, as shown in Fig. 2. Most wafer-scale methods are based on bottom-up strategies through chemical reactions using CVD, MOCVD, and ALD (Fig. 2a–f). With a traditional CVD method, wafer-scale uniformity can be achieved depending on the diameter of the quartz tube (2 and 4 inches), as shown in Fig. 2a and b. Through the CVD process, sulphur (S) and MoO_3_ sources are evaporated at high temperatures and delivered through tubes. By contrast, S gas is injected through a nozzle into a cylindrical chamber rather than a tubular chamber during MOCVD. The metal–organic source is generally Mo(CO)_6_ for Mo and (C_2_H_5_)_2_S and H_2_S for S owing to its high uniformity and easy control layer, as shown in Fig. 2c.^19^ In addition, Seol *et al.* achieved the largest size with a 6-inch wafer-scale monolayer of MoS_2_ and WS_2_ through pulsed MOCVD (Fig. 2d).^32^ ALD has advantages in terms of the precise control over large-scale wafers because of the self-limiting growth mechanism. Kim *et al.* also reported self-limiting layer synthesis (SLS), in which the number of layers can be controlled by temperature rather than by the process cycles of the ALD. The SLS can produce MoS_2_ with a uniformity of 90% and a wafer-scale (∼10 cm), as shown in Fig. 2e.^33^ Moreover, Jang *et al.* reported a Mo(CO)_6_ precursor and H_2_S plasma for a 4-inch wafer size at 175 °C to 225 °C (Fig. 2f). The process temperature of ALD is lower than that of CVD.^34^ Desai *et al.* reported gold-assisted exfoliation using the interaction between gold and sulphur; therefore, it has been used in the formation of self-assembled monolayers of thiolated organic molecules on gold surfaces (Fig. 2g). It can exfoliate a large monolayer area from the bulk TMD crystal.^35^ In addition, Li *et al.* reported the VLS growth of a uniform monolayer of MoS_2_ flakes on a 4-inch or continuous MoS_2_ film with a 100 μm grain on a 2-inch sapphire with a molten precursor (*e.g.* non-volatile Na_2_MoO_4_), as shown in Fig. 2h.^21^ Na_2_MoO_4_ was coated through spin-coating and melted at a temperature higher than its melting point (687 °C) at 750 °C. Recently, Chang *et al.* reported a self-capping VLS reaction for the growth of large single crystals and full-coverage TMD films, as shown in Fig. 2i.^36^ Ultra-thin MoO_3_, SiO_2_, and NaF layers were used for forming eutectic liquid (Na_2_Mo_2_O_7_) rising to the surface at high temperature. It was sulfurized to MoS_2_ seeds and acted as a self-capping layer. Moreover, a continuous MoS_2_ film can be fabricated using MoS_2_ seeds with a large grain size (∼450 μm). Finally, the thermolysis of thiosalts was conducted through the thermal decomposition of (NH_4_)_2_MoS_4_ at high temperatures. Lim *et al.* reported bar-coating on 50 cm Ni foil with (NH_4_)_2_MoS_4_ and thermally decomposed through the roll-to-roll process, as shown in Fig. 2j.^22^ It can achieve a long-range uniformity with an easy transfer to other substrates. In addition, Park *et al.* introduced a laser-selective synthesis for MoS_2_ or WS_2_ patterning under ambient conditions (Fig. 2k),^37^ which has an advantage for ultrafast and independent synthesis of TMDs. In contrast to the 1T phase, for which only limited synthesis methods have been reported, the 2H phase of TMDs and wafer-scale methods with various synthesis mechanisms have already been reported.

## 3. PECVD synthesis method for TMDs

### 3.1 TMD phase control synthesis using PECVD

PECVD has mainly been used for plasma etching or the synthesis of SiO_2_ and Si_3_N_4_ in semiconductor manufacturing. In 2015, we first succeeded in the synthesis of 2H-MoS_2_ using an inductively coupled plasma (ICP) type PECVD.^9^ At that time, a reactive ion etcher (RIE) machine for synthesis was converted with high-purity H_2_S gas (99.99%) and a moving chuck to control the height of the sheath. The synthesis conditions of 2H-MoS_2_ were optimised with many parameters such as the plasma power, chamber pressure, process time, chuck temperature, chuck height, gas species, gas ratio (Ar to H_2_S), and flow rate. After 5 years, Kim *et al.* found optimised conditions for 1T-WS_2_ in 2020.^10^ This is the first time that a wafer-scale 1T phase synthesis was achieved because the metastable nature of 1T-TMDs is challenging to fabricate and synthesise. We found a breakthrough for 1T-TMD synthesis using a PECVD system with Ar + H_2_S plasma. Moreover, we used the same ICP type PECVD under different conditions for the synthesis of both phases (1T and 2H). Kim *et al.* then succeeded in applying it to other TMDs for 1T-MoS_2_ in 2021;^11^ thus, MoS_2_ and WS_2_ can be synthesised through the 1T and 2H phases using the same PECVD.

**Fig. 3:**
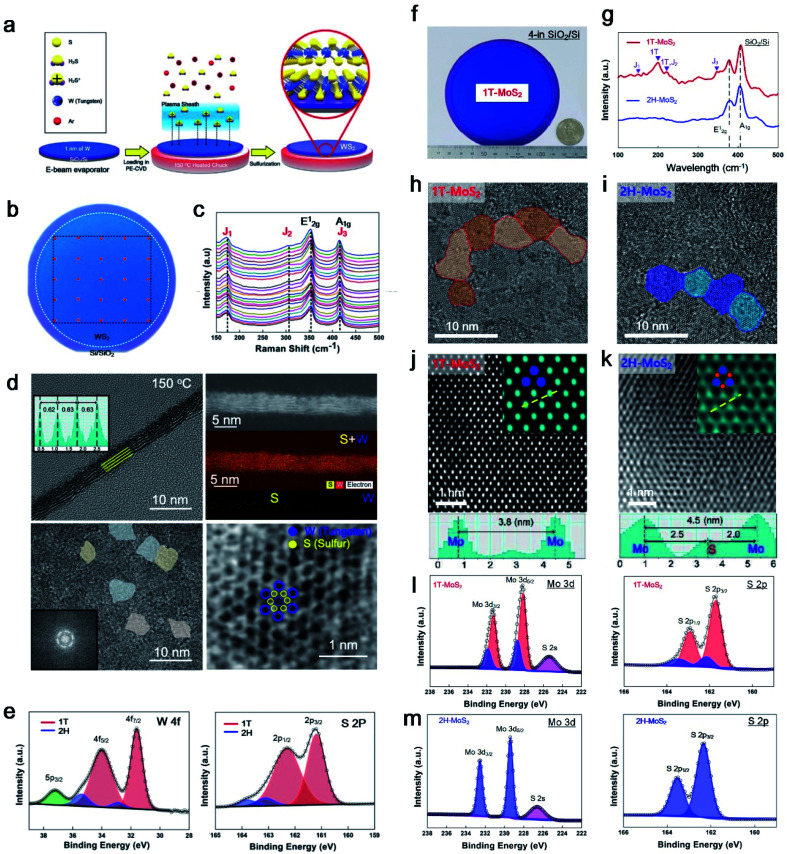
The unique properties of PECVD-grown MoS_2_ and WS_2_. (a) Synthesis sequence of 1T-WS_2_ through PECVD, (b) photograph of the 4-inch scale of 1T-WS_2_ with (c) 25 points in the Raman spectra, (d) HR-TEM images showing 4 layers of nano-grain octahedral (1T) structures, and (e) XPS profiles for W 4f and S 2p comprising 1T (red) and 2H (blue) peaks (reproduced with permission from ref. 10, copyright 2020 Wiley-VCH Verlag GmbH & Co). (f) Optical image of wafer-scale 1T-MoS_2_ with a quarter, (g) Raman spectra for comparing 1T and 2H-MoS_2_, (h and i) in-plane HR-TEM image and line profiling of phase-controlled MoS_2_ (1T or 2H) depending on plasma process temperature, (j and k) STEM image for the atomic structure of 1T and 2H-MoS_2_, (l) XPS profiles for 1T-MoS_2_ with Mo 3d and S 2p, and (m) for 2H-MoS_2_ with Mo 3d and S 2p (reproduced with permission from ref. 11, copyright 2021 Wiley-VCH Verlag GmbH & Co).

Fig. 3a shows in more detail the sequence of 1T-WS_2_ thin films, where 1 nm of W was deposited using an e-beam evaporator and sulfurization with Ar + H_2_S plasma at 150 °C. Owing to the low-temperature process, 1T-WS_2_ was successfully synthesised on arbitrary substrates (SiO_2_/Si wafer, glassy carbon electrode (GCE), Teflon, and PI). The photograph in Fig. 3b shows a 4-inch wafer of a 1T-WS_2_ thin film, as indicated in the blue area, and the uniformity of the 4-inch scale of 1T-WS_2_ was verified by the 25 points in the Raman spectra shown in Fig. 3c. The as-synthesised 1T-WS_2_ possesses 4–5 layers with nanograins, which expose abundant grain boundaries and edge sites working as catalytically active sites, as shown in Fig. 3d. An XPS analysis can be used to configure the 1T phase with the core peak regions of W 4f and S 2p (Fig. 3e). Similarly, Kim *et al.* demonstrated a 4-inch scale of 1T-MoS_2_ thin films using cold plasma and controlled the phases for octahedral (1T) and trigonal prismatic (2H) phases depending on the chuck temperature. Fig. 3f shows an image of the as-grown 1T-MoS_2_ film on a 4-in SiO_2_/Si substrate. The Raman spectra show two main peaks (E^1^_2g_ and A_1g_) from both 1T and 2H-MoS_2_; however, *J*_1_, *J*_2_, and *J*_3_, which correspond to the reduced symmetry of the 1T phase, were only observed for 1T-MoS_2_ in Fig. 3g. The synthesised 1T and 2H phase MoS_2_ has nanograins with different atomic structures, as confirmed through a high-annual dark-field (HAADF)-STEM in the [001] zone with line profiling on HR-TEM images, as shown in Fig. 3h–k. HAADF-STEM images provide an intuitively interpretable “Z-contrast” to distinguish Mo and S atoms, and thus it can distinguish a hexagonal lattice structure for 1T and a honeycomb lattice structure for 2H-MoS_2_, as shown in Fig. 3j–k. The deconvoluted XPS spectra of Mo 3d and S 2p of 1T-MoS_2_ were slightly shifted to lower binding energies than those of 2H-MoS_2_ in Fig. 3l–m. Therefore, Raman, XPS, and HR-TEM can clearly distinguish the two phases (1T and 2H) of the PECVD-grown MoS_2_.

### 3.2 Applications based on nanocrystalline MoS_2_ synthesised using PECVD

**Fig. 4:**
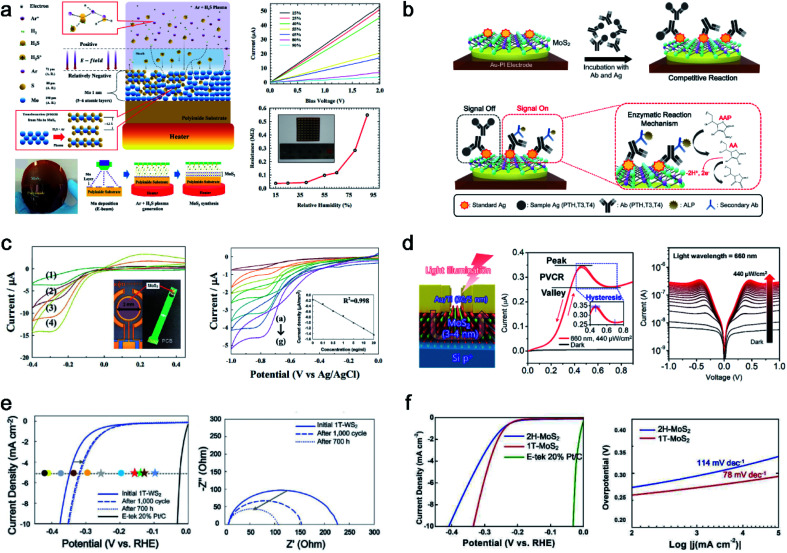
PECVD-grown nanocrystalline MoS_2_ with various applications: (a) PECVD synthesis mechanism for nanocrystalline MoS_2_ thin films with a humidity sensor array (reproduced with permission from ref. 9, copyright 2015 Wiley-VCH Verlag GmbH & Co), (b) direct fabrication of flexible MoS_2_-PI electrodes for PTH, T3, and T4 hormone detection (reproduced with permission from ref. 40, copyright 2020 American Chemical Society), (c) direct synthesis of a MoS_2_ thin film on a PCB for the observation of hydroquinone by immobilized IgG-HRP for H_2_O_2_ reduction (reproduced with permission from ref. 41, copyright 2015 Royal Society of Chemistry), (d) MoS_2_ on p-Si based negative differential resistance photodetectors with high uniformity and yield (reproduced with permission from ref. 43, copyright 2021 American Chemical Society), (e) electrocatalytic performance with the stability test of 1T-WS_2_ for LSV curves and EIS (reproduced with permission from ref. 10, copyright 2021 Wiley-VCH Verlag GmbH & Co), and (f) electrocatalytic performance comparison of 1T and 2H-MoS_2_ for LSV curves and Tafel plots (reproduced with permission from ref. 11, copyright 2020 Wiley-VCH Verlag GmbH & Co).

The MoS_2_ thin film was synthesized with unique properties by using the PECVD method. This results in nanocrystalline and nano-sized grain boundaries, whereas the micron size of the MoS_2_ film is common in conventional processes. Considering the plasma synthesis mechanism, radicals and ions are accelerated to the substrate for sulfurization, and ion bombardment causes a breakage of the Mo metal film for efficient chemical reactions. Unlike large grains through CVD synthesis, nanocrystalline MoS_2_ is outstanding in fields that require many defects or grain boundaries. Herein, we applied various applications for humidity sensors, flexible biosensors, photodetectors, and the hydrogen evolution reaction (HER), as shown in Fig. 4. Ahn *et al.* successfully synthesised a 4-inch scale MoS_2_ thin film directly on a polyimide (PI) substrate at low temperature (150 °C) and demonstrated MoS_2_ humidity sensors, as shown in Fig. 4a.^9^ This is the first study conducted on the Mo metal sulfurization mechanism in an inductively coupled plasma (ICP) system. The 1 nm pre-deposited Mo metal was sulfurized using H_2_S^+^ and Ar^+^, which can react with Mo at low temperatures, resulting in 5 or 6 layers of MoS_2_ thin films. The ionised H_2_S in the plasma (positive) is accelerated to the substrate (relatively negative) by the electric field in the sheath region, and Ar helps ionise H_2_S through the Penning effect with an optimised ratio. The ion bombardment of H_2_S^+^ can penetrate the Mo metal layer for sulfurization to synthesise MoS_2_. This process enabled the direct synthesis of MoS_2_ thin films on flexible plastic substrates. They also demonstrated MoS_2_ thin-film-based flexible humidity sensor arrays on PI substrates, which were measured based on the *I*_DS_–*V*_DS_ characteristics (resistance change) at different relative humidity (RH) from 15% to 95%.

To take advantage of the low-temperature process, H.-U. Kim *et al.* directly synthesised MoS_2_ on an Au patterned polyimide (PI) substrate (MoS_2_-Au-PI) as a flexible electrode for a biosensor (Fig. 4b).^40^ PECVD can fabricate twelve MoS_2_-Au-PI electrodes with high uniformity and reproducibility at 150 °C. The fabricated MoS_2_-Au-PI flexible biosensors can provide sufficient active sites owing to the nano-grain structures. It can detect three types of hormones (T3, T4, and PTH) with their respective AAP substrates, as confirmed through cyclic voltammetry (CV) measurements at various concentrations. The fabricated ALP-(PTH, T3, T4)-MoS_2_-Au-PI flexible complex was investigated with different concentrations of PTH, T3, and T4. The calibration plot between the oxidation peak current and the different standard materials of PTH, T3, and T4 are observed at 0.38 eV, showing good reproducibility and sensitivity.

Owing to its compatibility with an arbitrary substrate for the synthesis of the PECVD system, MoS_2_ can be synthesised on polymeric printed circuit boards (PCBs), as shown in Fig. 4c.^41^ The negative charges carried by MoS_2_ are used to immobilize IgG-HRP through electrostatic attraction. The designed MoS_2_-HRP electrode was operated for H_2_O_2_ reduction to observe hydroquinone (HQ). The anodic and cathodic peak currents at the MoS_2_-HRP electrode were investigated using CV, depending on the scan rate. Depending on the IgG-HRP concentration ranging from 0 to 20 ng mL^−1^, the reduction current was linear with a correlation coefficient of 0.998. Owing to the MoS_2_ thin films having excellent electrocatalytic characteristics, the electron transfer between the HRP and underlying electrode can be enhanced. An *in situ* synthesis of MoS_2_ on a PCB was used for enzyme immobilization of a biocompatible matrix for a highly sensitive electrochemical biosensing platform.

Woo *et al.* transferred PECVD-grown MoS_2_ onto a p-Si wafer through a residue-free water transfer method^42^ for negative differential resistance (NDR) photodetectors, as shown in Fig. 4d.^43^ The MoS_2_/p-Si heterostructures provided a 100% yield, high uniformity, and robust NDR photodetectors for a sub-1-volt operation. The potential well between MoS_2_ and p-Si is modulated owing to the increased Fermi level of MoS_2_ under illumination, resulting in a light-induced NDR effect. The 120 devices showed a 100% yield of NDR behaviour under illumination at five different wavelengths (420, 455, 530, 660, and 1050 nm). The fabricated NDR devices show a peak current of 0.35 μA at a low operating voltage of 0.45–0.7 V under a 660 nm wavelength with 440 μW cm^−2^. The uniformity and electrical performance of 120 NDR devices were characterized by a peak-to-valley current ratio of 1.195 ± 0.0065. Furthermore, the designed NDR devices showed an unprecedented robustness for 100 000 cycles without failure under illumination.

Kim *et al.* confirmed through HR-TEM that PECVD-grown WS_2_ is nanocrystalline and that nanograin-boundary activations during cycles induce an HER performance enhancement (from 347 mV at 5 mA cm^−2^ initially to 315 mV at 5 mA cm^−2^ after 1000 cycles of the HER and 700 h), as shown in Fig. 4e.^10^ Moreover, the nanocrystalline structure of 1T-WS_2_ makes a meta-stable to highly stable structure with a lower surface energy than the 2H structure, and thus 1T-WS_2_ preserves its 1T structure even after 1000 cycles of the HER. Similarly, Kim *et al.* reported that another 1T-TMD (1T-MoS_2_) synthesized by PECVD was also tested for 1000 cycles of the HER as shown in Fig. 4f,^11^ exhibiting a stable 1T structure. Finally, the PECVD method is the first approach for both the 1T and 2H phase synthesis methods, while controlling the temperature and other parameters. To compare the intrinsic catalytic performance, HER measurements were conducted for both phases of MoS_2_. 1T-MoS_2_, which has a high density of catalytically active sites, shows an enhanced HER performance (0.29 V at 5 mA cm^−2^) in comparison to 2H-MoS_2_ (0.33 V at 5 mA cm^−2^). The corresponding Tafel slopes of 1T- and 2H-MoS_2_ were 78 mV decade^−1^ and 114 mV decade^−1^, respectively. After 1000 cycles of the HER, 1T-MoS_2_ preserves the 1T phase, as confirmed through Raman and XPS; thus, PECVD-grown 1T-MoS_2_ can have a stable structure even under harsh conditions.

### 3.3 PECVD based synthesis of heterostructures with applications

**Fig. 5:**
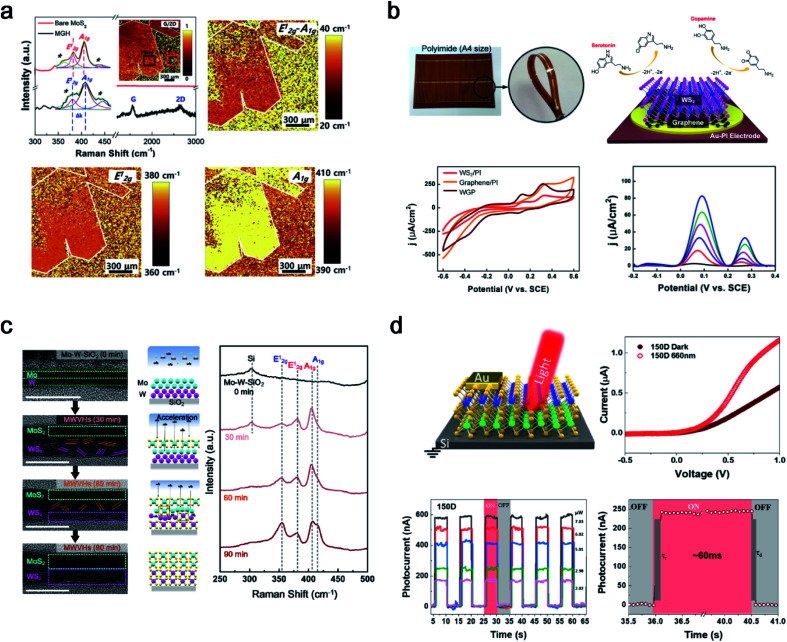
2D heterostructures synthesised using PECVD. (a) Raman spectrum with large-scale Raman mapping images of the PECVD grown 2H-MoS_2_/graphene heterostructure (reproduced with permission from ref. 44, copyright 2019 Elsevier), (b) WS_2_/graphene heterostructures under Au-polyimide (WGP) fabrication and a flexible electrochemical sensor for simultaneous detection of dopamine and serotonin (reproduced with permission from ref. 45, copyright 2021 Wiley-VCH Verlag GmbH & Co), (c) 2H-MoS_2_/2H-WS_2_ vertical heterostructure synthesis with penetrative H_2_S plasma with mechanism investigation through a time-dependent analysis (reproduced with permission from ref. 46, copyright 2021 American Chemical Society), and (d) 2H-MoS_2_/2H-WS_2_ photodetector on a boron-doped Si wafer (reproduced with permission from ref. 47, copyright 2021 American Chemical Society).

The layer-by-layer stacking of atomically thin 2D heterostructures was intensively studied owing to their unique features in various areas. After successful 2D TMD thin film synthesis with cold plasma, PECVD was extended to heterostructures between 2D thin films, such as graphene and TMDs (MoS_2_ and WS_2_). The first approach was a heterostructure between graphene and a 2H-MoS_2_ thin film as shown in Fig. 5a.^44^ Although the fabrication of a wafer-scale with highly uniform 2D heterostructures is challenging, the first success of wafer-scale 2H-MoS_2_/graphene heterostructures (MGH) using Ar + H_2_S plasma was reported. After transferring CVD-grown graphene onto the substrate, Mo metal seed layer deposition and further sulfurization in PECVD were conducted. Owing to the lattice mismatch between 2H-MoS_2_ and graphene, dense defects and sulphur (S) vacancies were generated, as verified by large-area Raman spectral mapping. The generated S vacancy was observed in a low stoichiometry of 1.78 from XPS and an S vacancy peak at 448 cm^−1^ from Raman spectroscopy. The enhanced catalytic activity in MGH was due to the synergistic effect of dense defects and S vacancies for active sites and the efficient charge transport in highly conductive graphene layers. Furthermore, 1T-WS_2_/graphene heterostructures on Au-polyimide (WGP) were designed for concurrent and selective determination of dopamine and serotonin, as shown in Fig. 5b.^45^ Owing to the low-temperature process using cold plasma, direct growth of a 1T-WS_2_/graphene heterostructure on a flexible substrate was demonstrated for the first time. This overcomes the limitations of conventional heterostructures (small-sized heterostructures, poor uniformity and reproducibility, and high-temperature process) with a 4-inch size and uniform quality at 150 °C. The as-fabricated WGP shows a highly sensitive and selective electrochemical performance with well-separated voltammetric signals for dopamine and serotonin with a potential gap of 188 mV. Differential pulse voltammetry (DPV) was conducted to investigate the catalytic activity toward dopamine and serotonin. The WGP shows the simultaneous determination of dopamine ((1.24–6.17) × 10^−6^ M) and serotonin (249 × 10^−9^ to 4.95 × 10^−6^ M) in PBS (0.1 M; pH = 7.5). The anodic peak current density increased linearly with the concentration of dopamine and serotonin, with high correlation coefficients of 0.996 and 0.991, respectively. In addition, after 30 days, the WGP sensors preserved 83.7% and 82.5% of their initial responses toward dopamine and serotonin, respectively. The PECVD system can innovate conventional methods and fabricate highly sensitive and robust biosensors.

TMD–TMD heterostructures (TTHs) are promising candidates for future semiconductors owing to their interesting properties such as tunnelling, bandgap alignment, and interlayer coupling. In this regard, synthesis methods for TTHs are an attractive area for materials scientists. However, there are some hurdles to overcome, such as the two-step annealing process, small-sized heterointerfaces with non-uniformity, and overlapping structures. Seok *et al.* reported a novel process that can overcome all hurdles using a penetrative plasma process. Double metal seed layers of W (1 nm) and Mo (1 nm) were sequentially deposited on the substrate, and the amorphous phase of the Mo–W layers was sulfurized to 2H-MoS_2_/2H-WS_2_ vertical heterostructures (MWVHs) in Ar + H_2_S plasma at 300 °C (Fig. 5c).^46^ Similar to the 2H-MoS_2_ synthesis mechanism, ion bombardment of H_2_S^+^ can penetrate the Mo–W metal layer in a one-step process and was investigated using HRTEM and a Raman analysis in a time-dependent manner. In addition, a reverse order (W–Mo) of the metal layers or various thicknesses of Mo–W were applied and successfully investigated to synthesise 2H-WS_2_/2H-MoS_2_ vertical heterostructures (WMVHs) or MWVHs with different thicknesses. The as-synthesised MWVHs were evaluated for interfacial interaction between MWVHs and SiO_2_/Si at 1.14 ± 0.14 J m^−2^ using large double cantilever beam (DCB) specimens, confirming robust van der Waals interactions between MoS_2_ and WS_2_. In addition, 225 MWVH diodes were fabricated to confirm the wafer-scale uniformity. A superior electrical performance was achieved compared to the separated-step and transferred MWVHs. This study provides insight into a general method for wafer-scale 2D heterostructures using single-step penetrative plasma. In addition, MWVHs were fabricated on SiO_2_/Si and transferred onto boron-doped Si for application as a photodetector, as shown in Fig. 5d.^47^ Two as-fabricated MWVH photodetectors were prepared at 150 °C (150D), and MWVHs were further annealed at 300 °C (300D). The photocurrent generation at 150D under light illumination of wavelengths (*λ* = 420, 530, and 660 nm) is larger than that at 300D except in the case of 1050 nm. Owing to the heterojunction between 2H-MoS_2_ and 2H-WS_2_, the 150D photodetector showed a better performance than 2H-MoS_2_ or 2H-WS_2_ individually. Therefore, a TTH was successfully fabricated in a one-step process with PECVD and applied to both diodes and photodetectors.

## 4. Conclusions and outlook

In this review, a summary of the recent highlights of the 1T phase of TMDs based on their synthesis methods and limitations and the progress of PECVD based TMD synthesis and their applications is provided. TMDs have unique properties as polymorphs depending on the phases, such as 1T (metal), 1T′ (semimetal-like), and 2H (semiconductor) phases; thus, various synthesis methods have been reported for each phase. However, the 1T structure is meta-stable, and only limited synthesis methods have been reported. Despite the remarkable advances in the literature, a new synthetic method for 1T-TMDs using PECVD with innovative challenges is introduced. Compared with the high-temperature process by using CVD (550–1000 °C), ionized gases in cold plasma can dramatically decrease the process temperature (150–300 °C). The first progress of cold plasma-based TMD synthesis with unique properties can be introduced.

(1) Depending on the process temperature, a controlled polymorph of TMDs can be synthesized by optimized plasma characteristics. The 1T phase of MoS_2_ and WS_2_ was synthesised as a 4-inch wafer-scale thin film for the first time, and the process temperature was 150 °C and the 2H phase was synthesised at 300 °C. The phases of the TMDs were distinguished using STEM, Raman spectroscopy, and XPS.

(2) PECVD-grown TMDs have unique properties such as nano-grains, which expose abundant grain boundaries and edge sites working as catalytically active sites, and a high uniformity on a 4-inch wafer scale; thus, it has advantages for HER or biosensor applications with active sites following the use of nanograins. Moreover, the nanocrystalline structure of 1T-WS_2_ forms a meta-stable to highly stable structure with a lower surface energy than the 2H structure; thus, 1T-WS_2_ preserves its 1T structure even after 1000 cycles of the HER.

(3) The PECVD-based 2D-TMD synthesis process has been extended to 2D heterostructures such as TMDs–graphene or TMD–TMD. The PECVD method can synthesise TMD heterostructures even on a graphene-transferred SiO_2_/Si substrate. Furthermore, double metal seed layers of W (1 nm) and Mo (1 nm) were sequentially deposited on the substrate, and the amorphous phase of the Mo–W layers was sulfurized to 2H-MoS_2_/2H-WS_2_ vertical heterostructures with a single-step penetrative plasma mechanism.

Overall, the first progress of plasma based TMD synthesis is introduced in this review to give insight into advanced 2D material synthesis. It overcomes the limitation of the conventional synthesis method by wafer-scale, high uniformity, and grain and heterointerface modulation, which can be advantageous for various applications.

## Conflicts of interest

There are no conflicts to declare.

## Supplementary Material
